# Multiplex RPA-CRISPR/Cas12a Assay for Rapid Detection of Class D OXA-Type Carbapenem-Resistant *Acinetobacter baumannii*

**DOI:** 10.3390/bios16080422

**Published:** 2026-08-05

**Authors:** Meruyert Amanzholova, Ainur Akimbekova, Aisha Shaizadinova, Nazgul Sutimbekova, Nelya Bissenova, Pavel Tarlykov, Sailau Abeldenov

**Affiliations:** 1 National Center for Biotechnology, Astana 010000, Kazakhstan; 2 Faculty of Biology and Biotechnology, Farabi Kazakh National University, Almaty 010000, Kazakhstan; 3 Department of Microbiology and Virology, Astana Medical University, Astana 010000, Kazakhstan; 4 National Scientific Medical Center, Astana 010000, Kazakhstan

**Keywords:** *Acinetobacter baumannii*, carbapenem resistance, *bla_OXA-23_*, *bla_OXA-40_*, CRISPR/Cas12a, recombinase polymerase amplification (RPA), multiplex detection, antimicrobial resistance, rapid diagnostics, point-of-care testing

## Abstract

*Acinetobacter baumannii* is a critical WHO priority pathogen due to its multidrug resistance and high mortality in carbapenem-resistant infections. Resistance is predominantly mediated by class D carbapenemase genes *bla*_OXA-23_ and *bla*_OXA-40_, which spread rapidly via horizontal gene transfer in healthcare settings. To address the lack of a rapid assay capable of detecting both blaOXA-23 and blaOXA-40 in a single analytical workflow, we developed a multiplex two-step RPA–CRISPR/Cas12a assay. Since infections caused by strains harboring either gene require identical therapeutic management, their co-detection in a single reaction is clinically justified. Although simultaneous use of two crRNAs within a single CRISPR/Cas12a reaction is often considered technically challenging due to potential inter-crRNA competition, here it advantageously enables dual-target coverage without compromising sensitivity. The assay demonstrated high specificity with no cross-reactivity against a panel of clinically relevant bacterial species, including closely related *Acinetobacter* spp. Evaluation using genomic DNA extracted from 63 cultured clinical *A. baumannii* isolates revealed *bla*_OXA-23_ in 19 isolates (30.2%), *bla*_OXA-40_ in 28 (44.4%), and co-carriage of both genes in 9 (14.3%), with at least one resistance gene detected in 60.3% of isolates. The complete workflow was accomplished within 45 min without specialized equipment, offering a rapid, sensitive, and cost-effective solution for point-of-care molecular surveillance of carbapenem-resistant *A. baumannii* in clinical and resource-limited settings.

## 1. Introduction

Antimicrobial resistance (AMR) is recognized as one of the most significant threats to global public health, healthcare systems, and socio-economic development [1,2,3]. According to global estimates, infections caused by resistant pathogens are responsible for more than 1.27 million deaths annually, with up to 4.95 million deaths associated with AMR worldwide [4]. Projections suggest that, if current trends persist, AMR-related mortality may reach 10 million deaths per year by 2050, exceeding mortality from cancer [5]. In addition to the clinical burden, AMR significantly increases healthcare costs due to prolonged hospital stays, the need for last-resort antibiotics, and reduced effectiveness of surgical procedures and chemotherapy [6].

A substantial proportion of this burden is associated with healthcare-associated infections (HAIs), which are predominantly caused by multidrug-resistant (MDR) pathogens capable of persisting in hospital environments. Particular attention has been given to the ESKAPE group of pathogens—*Enterococcus faecium*, *Staphylococcus aureus*, *Klebsiella pneumoniae*, *Acinetobacter baumannii*, *Pseudomonas aeruginosa*, and *Enterobacter* spp. which are characterized by their ability to “escape” antimicrobial action through diverse resistance mechanisms, including enzymatic degradation, target modification, and active efflux [7,8,9]. These organisms are responsible for a large proportion of severe nosocomial infections, including pneumonia, bacteremia, and urinary tract infections. In its updated Bacterial Priority Pathogens List (BPPL 2024), the World Health Organization classified carbapenem-resistant *A. baumannii* as a critical priority pathogen—the highest tier among 15 antibiotic-resistant bacterial groups—owing to the urgent need for new therapeutic and diagnostic strategies [10].

*A. baumannii* has emerged as one of the most clinically significant and problematic pathogens, particularly in intensive care units [11,12,13]. This organism is associated with ventilator-associated pneumonia, bloodstream infections, wound infections, and urinary tract infections, especially in immunocompromised patients [14,15]. Carbapenem-resistant *A. baumannii* (CRAB) is associated with particularly high mortality: reported case fatality rates among critically ill ICU patients range from 40% to 70%, compared to approximately 25% for carbapenem-susceptible strains [16,17]. Notably, carbapenem-resistant *A. baumannii* was ranked among the top pathogen–drug combinations for AMR-attributable deaths globally, with an estimated 57,700 deaths directly attributable to CRAB in 2019 alone [10]. Beyond mortality, CRAB infections impose a substantial economic burden: compared to carbapenem-susceptible infections, CRAB is associated with an excess hospital length of stay of up to 15.8 days and significantly higher total hospitalization costs, placing additional strain on already resource-limited healthcare systems [18]. Its success as a nosocomial pathogen is attributed to its remarkable ability to survive desiccation, form biofilms on abiotic surfaces, and rapidly acquire resistance determinants through horizontal gene transfer, frequently mediated by plasmids and mobile genetic elements [19,20,21,22].

Clinically, *A. baumannii* strains are classified based on their resistance profiles into multidrug-resistant (MDR), extensively drug-resistant (XDR), and pandrug-resistant (PDR) categories, reflecting increasing levels of therapeutic difficulty [23,24]. Of particular concern is resistance to carbapenems, which are considered last-resort antibiotics for severe infections [25,26,27]. This resistance is primarily mediated by carbapenem-hydrolyzing class D β-lactamases (CHDLs), also known as OXA-type enzymes [28,29,30]. Among them, intrinsic OXA-51-like enzymes are present in all *A. baumannii* strains but typically exhibit low expression, whereas acquired carbapenemases such as OXA-23-like, OXA-24/40-like, and OXA-58-like play a major role in clinically relevant resistance [31,32,33]. In particular, the OXA-23-like group is widespread all over the world and frequently associated with mobile genetic elements, facilitating rapid dissemination in hospital settings [34]. Carbapenem resistance has been reported at critically high levels in clinical *A. baumannii* isolates in some Central Asian settings, reflecting a broader global trend that has prompted the WHO to designate carbapenem-resistant *A. baumannii* as a critical priority pathogen and to promote AMR surveillance networks such as CAESAR [35].

Given the critical role of these resistance determinants, rapid and accurate detection of *A. baumannii* and its associated resistance genes is essential for effective infection control and targeted antimicrobial therapy [36,37,38,39]. Conventional microbiological methods, including culture-based identification and phenotypic susceptibility testing, remain the gold standard but are inherently time-consuming, typically requiring 24–72 h to yield results [40,41]. Moreover, these methods do not directly identify the underlying genetic mechanisms of resistance.

Molecular techniques such as polymerase chain reaction (PCR) and quantitative PCR (qPCR) have significantly improved diagnostic sensitivity and specificity, allowing direct detection of resistance genes, including blaOXA variants [42]. Their widespread implementation is limited by the need for expensive instrumentation, thermal cycling, and trained personnel. In addition, many existing assays are single-target, increasing the risk of false-negative results in the presence of genetic variability or multiple resistance mechanisms [43].

In recent years, isothermal amplification methods have emerged as promising alternatives to conventional PCR-based diagnostics, offering rapid nucleic acid amplification at a constant temperature without the need for thermal cycling equipment. Among these, recombinase polymerase amplification (RPA) has gained particular attention due to its ability to amplify target sequences within 10–20 min at 37–42 °C using a recombinase-primer complex to facilitate strand invasion and DNA synthesis [44], making it especially attractive for point-of-care applications in resource-limited settings. CRISPR/Cas12a further enhances this diagnostic potential by providing a programmable, sequence-specific recognition layer downstream of amplification: upon crRNA-guided binding to its target, Cas12a undergoes conformational activation and acquires collateral cleavage activity, indiscriminately cleaving single-stranded DNA reporter molecules and generating a measurable fluorescent signal [45,46,47,48]. This trans-cleavage mechanism functions as a built-in signal amplification step, substantially enhancing analytical sensitivity and specificity, and has been successfully applied for the detection of various pathogens and resistance genes [49,50,51,52,53,54]. The integration of RPA with CRISPR/Cas12a thus combines the speed and simplicity of isothermal amplification with the programmable specificity of CRISPR technology, resulting in a powerful diagnostic platform compatible with point-of-care implementation.

Despite significant progress, most currently available RPA–CRISPR systems remain single-target or limited in multiplexing capacity, which reduces their robustness in the context of genetically diverse pathogens such as *A. baumannii* [55,56]. Single-target assays are particularly vulnerable to sequence variability, including mutations and deletions in target regions, which may result in false-negative outcomes. Moreover, *A. baumannii* isolates frequently harbor multiple carbapenemase genes simultaneously, with regional variation in the prevalence of OXA-23 and OXA-24/40-like groups [57]. To address this diagnostic gap, the present study employed a multiplexed RPA-CRISPR/Cas12a approach for the simultaneous detection of *bla_OXA-23_* and *bla_OXA-40_*, the two most prevalent acquired class D carbapenemases in *A. baumannii*. This approach proved clinically relevant, as simultaneous carriage of both resistance genes was identified in 9 of 63 clinical isolates (~14%), a finding consistent with reports of co-occurring acquired blaOXA-type carbapenemases in *A. baumannii* from diverse geographic regions [20,57]. Single-target assays targeting only one of these genes would inevitably underestimate the true prevalence of resistance and miss clinically significant co-resistance patterns.

Unlike many previously reported RPA–CRISPR diagnostic platforms, where multiplex amplification is followed by separate CRISPR/Cas reactions for each target or where multiple crRNAs are designed for different regions of a single gene [55], the system developed in this study combines multiplex RPA with simultaneous CRISPR/Cas12a detection using individual crRNAs specific for each resistance determinant within a unified workflow. Furthermore, to our knowledge, no previously published CRISPR-based diagnostic system has simultaneously targeted *bla_OXA-23_* and *bla_OXA-40_* within a single multiplexed assay, representing a distinct gap that the present work addresses. Such an approach enables concurrent identification of multiple carbapenem resistance genes in a single analytical pipeline while preserving high specificity and sensitivity. This design not only simplifies the diagnostic procedure and reduces reagent consumption, but also improves the robustness of detection in genetically diverse clinical isolates of *A. baumannii*.

In this context, the development of multiplex diagnostic systems capable of simultaneously detecting multiple resistance determinants represents a critical advancement. In the present study, we developed and evaluated a multiplex RPA-CRISPR/Cas12a system for the simultaneous detection of two key carbapenem resistance genes, *bla_OXA-23_* and *bla_OXA-40_*. The selection of these two targets is justified by their status as the predominant acquired class D carbapenemases in *A. baumannii* globally, collectively accounting for the majority of clinically relevant carbapenem resistance. This approach enhances diagnostic reliability by reducing the risk of false-negative results associated with genetic variability and provides a more comprehensive assessment of the resistance profile of clinical isolates. Furthermore, the proposed method retains the advantages of isothermal amplification, including rapid turnaround time, simplicity, and potential applicability in resource-limited and point-of-care settings.

## 2. Materials and Methods

**Figure 1:**
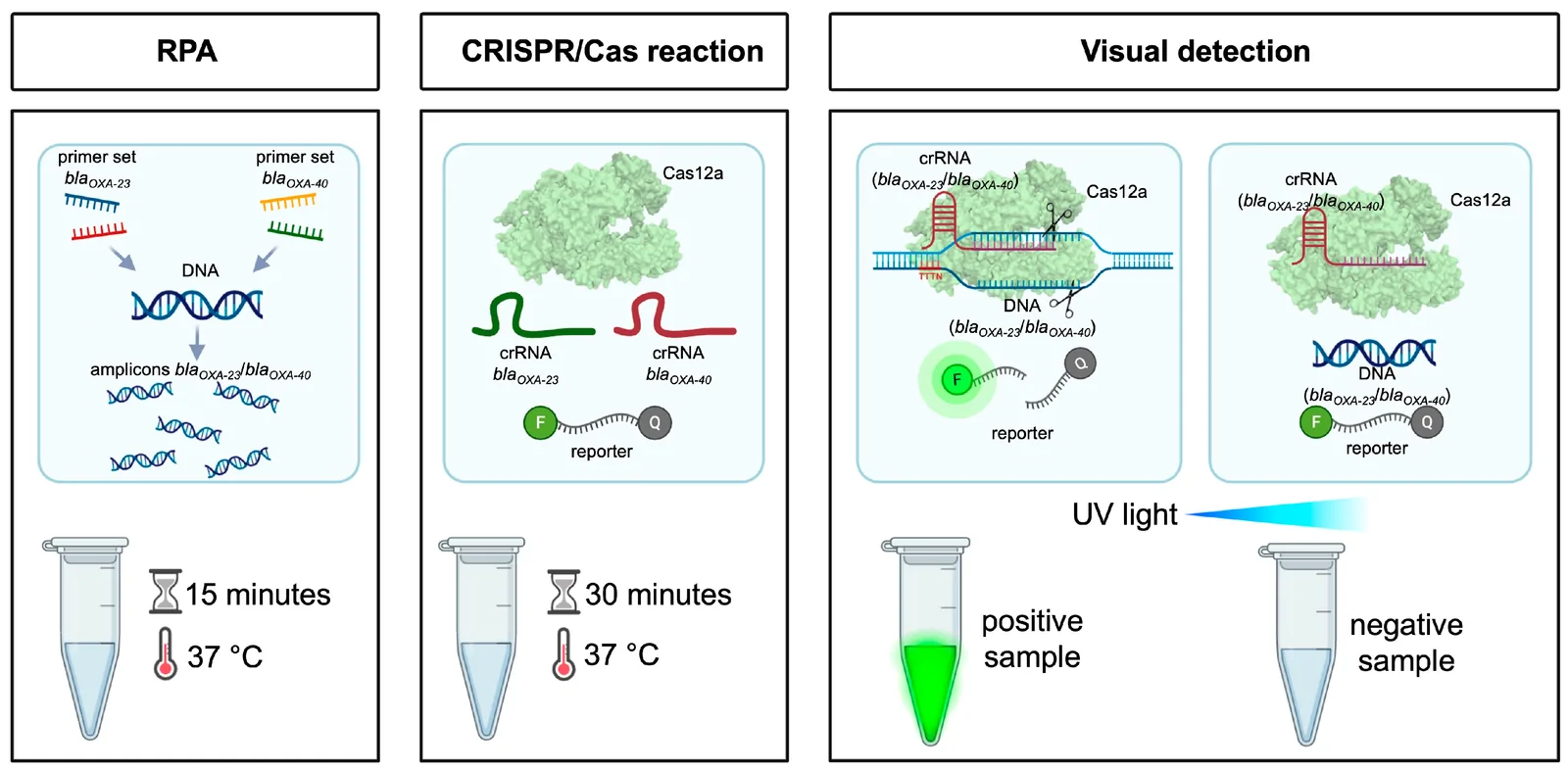
Schematic overview of the multiplex RPA-CRISPR/Cas12a workflow for detection of carbapenem-resistant *A. baumannii*. (1) Multiplex RPA of *bla_OXA-23_* and *bla_OXA-40_* (37 °C, 15 min); (2) CRISPR/Cas12a detection with two gene-specific crRNAs and a fluorophore-quencher reporter (37 °C, 30 min); (3) readout via fluorescence under UV light or real-time fluorometry. Total time: ~45 min.

To illustrate the conceptual framework of the proposed approach, a schematic overview of the diagnostic workflow is presented in Figure 1.

The workflow comprises three sequential stages. In the first stage, RPA, two primer sets targeting *bla_OXA-23_* and *bla_OXA-40_* are used for simultaneous isothermal amplification of both genes from genomic DNA at 37 °C for 15 min, generating target-specific amplicons. In the second stage, the CRISPR/Cas12a reaction, RPA products are incubated with Cas12a protein programmed with two gene-specific crRNAs targeting *bla_OXA-23_* and *bla_OXA-40_* in the presence of a fluorophore-quencher reporter at 37 °C for 30 min. Upon recognition of the target sequence, Cas12a is activated and cleaves the reporter molecule. In the third stage, visual detection, the activated Cas12a non-specifically cleaves the reporter molecules, releasing the fluorophore and generating a fluorescence signal visible under UV light: positive samples produce a bright green fluorescent signal, while negative samples remain non-fluorescent.

### 2.1. Bacterial Strains and Genomic DNA Extraction

Genomic DNA from the following bacterial species was used in this study: *Acinetobacter baumannii*, *Staphylococcus aureus*, *Escherichia coli*, *Pseudomonas aeruginosa*, *Salmonella enterica*, *Klebsiella pneumoniae*, *Streptococcus agalactiae*, *Acinetobacter nosocomialis*, *Enterobacter ludwigii*, *Staphylococcus haemolyticus*, *Acinetobacter pittii*, and *Acinetobacter junii*. Genomic DNA was extracted using the Monarch^®^ Spin gDNA Extraction Kit (NEB, Ipswich, MA, USA, T3010S) and the GeneJET Genomic DNA Purification Kit (Thermo Scientific, Waltham, MA, USA, K0721) according to the manufacturers’ instructions. All DNA samples were stored at −20 °C until further use.

### 2.2. RPA Primer Design

The nucleotide sequences of the target genes *bla_OXA-23_* and *bla_OXA-40_* were obtained from whole-genome sequencing data of the studied isolates. RPA primers were designed using Vector NTI v11 software (Invitrogen, Carlsbad, CA, USA) in accordance with the manufacturer’s guidelines for RPA reagent kits (TwistDX, Cambridge, UK). Primer variants were selected based on their specificity and efficiency of target gene amplification. For amplification of the *bla_OXA-23_* gene, the following primers were used: forward, 5′-AAGGTCATTTACCGCTTGGGAAAAAGACAT-3′; reverse, 5′-TGGTTTTATATCCATTGCCCAACCAGTCTTTCCA-3′. For amplification of the *bla_OXA-40_* gene, the following primers were applied: forward, 5′-TACACCAGTACAAGAAGTTAATTTTGCCGATGACC-3′; reverse, 5′-AGTAACATCCATTCCCCATCCACTTTTTGC-3′.

### 2.3. crRNA Design, Synthesis and Purification

**Figure 2:**
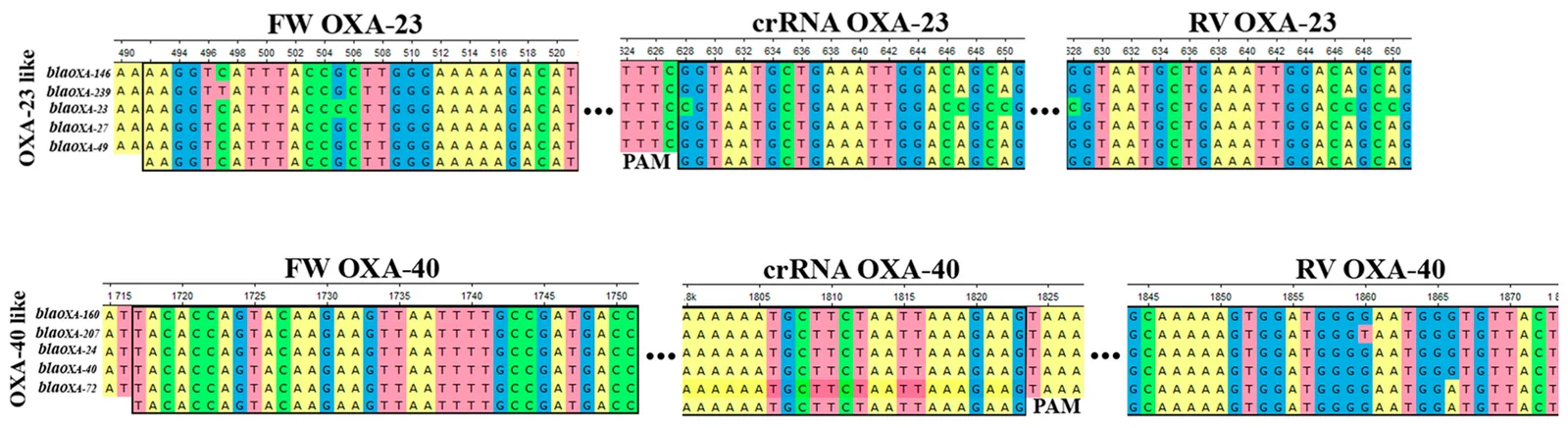
Alignment of the target regions showing the binding sites of the RPA primers and crRNAs used in the multiplex RPA–CRISPR/Cas12a assay. The positions of the forward (FW) and reverse (RV) RPA primers are indicated at the ends of each target region, while the crRNA target sequences are shown between the primer-binding sites. The protospacer adjacent motif (PAM) required for Cas12a recognition is highlighted in yellow. The **upper panel** corresponds to the *bla_OXA-23_* target region, and the **lower panel** corresponds to the *bla_OXA-40_* target region.

crRNA sequences targeting the RPA-amplified regions were designed using Vector NTI v11, with particular attention to the location of protospacer adjacent motif (PAM) sequences required for Cas12a nuclease activation. The PAMs TTTC and TTTA were selected for *bla_OXA-23_* and *bla_OXA-40_*, respectively. The oligonucleotides used as templates for crRNA generation were crRNA_OXA_23_TTTC_compl (5′-CTGCTGTCCAATTTCAGCATTACCATCTACAAACAGTAGAAATTCCCTATAGTGAGTCGTATTAGAATT-3′) and crRNA_OXA_40_TTTA_compl (5′-AAAAAATGCTTCTAATTAAAGAAGATCTACAAACAGTAGAAATTCCCTATAGTGAGTCGTATTAGAATT-3′). The locations of the RPA primer-binding sites, crRNA target sequences, and the corresponding (PAM) within the *bla_OXA-23_* and *bla_OXA-40_* target regions are shown in Figure 2. crRNA synthesis was performed by in vitro transcription using the HiScribe^®^ T7 High Yield RNA Synthesis Kit (New England Biolabs, Ipswich, MA, USA) according to the manufacturer’s protocol.

Following synthesis, crRNA was purified using the Monarch^®^ Spin RNA Cleanup Kit (New England Biolabs, Ipswich, MA, USA). The concentration of the purified crRNA was determined spectrophotometrically using a NanoDrop One instrument (Thermo Scientific, Waltham, MA, USA). The purified crRNAs were subsequently used for the assembly of ribonucleoprotein (RNP) complexes with Cas12a.

### 2.4. RPA

Recombinase polymerase amplification (RPA) was performed using a TwistDX kit (TwistDX Ltd., Cambridge, UK). For multiplex RPA, two primer sets targeting *bla_OXA-23_* and *bla_OXA-40_* were used, each at a concentration of 20 µM, with 1.2 µL of each forward and reverse primer added per reaction. The total reaction volume (50 µL) consisted of 29.5 µL rehydration buffer, 13.2 µL DEPC-treated water, 1.2 µL of each primer (for both primer sets), and 2.5 µL magnesium acetate. The reaction mixture was aliquoted, and genomic DNA was added as a template. Incubation was carried out at 37 °C for 15 min, followed by enzyme inactivation at 95 °C for 5 min. Amplification products were analyzed by agarose gel electrophoresis.

### 2.5. CRISPR/Cas12a Detection Assay

Recombinant *Moraxella bovoculi* Cas12a (MbCas12a) was produced and purified in-house at the National Center for Biotechnology (Astana, Kazakhstan) according to our previously published protocol. Briefly, the protein was expressed in *Escherichia coli* ArcticExpress (DE3) cells and purified by immobilized metal affinity chromatography followed by heparin affinity chromatography. Protein purity was verified by SDS-PAGE, and the nuclease activity of MbCas12a was confirmed as previously described [51].

CRISPR/Cas12a-based detection was performed using MbCas12a and corresponding crRNA at final concentrations of 100 nM each to form ribonucleoprotein complexes. The RPA product and a fluorescent reporter (final concentration 10 µM) were then added to the reaction mixture. The reaction was incubated at 37 °C, and Cas12a activation was assessed based on the generation of a fluorescence signal. Fluorescence was visualized under UV illumination and documented using a smartphone camera, captured at the reaction endpoint (30 min), as well as monitored in real time.

### 2.6. Real-Time Fluorescence Detection

Real-time fluorescence measurements were performed using a CFX96 system (Bio-Rad, Hercules, CA, USA). Fluorescence signals were recorded at 50-s intervals, with a 10-s acquisition time per cycle. Data were analyzed using the instrument software and GraphPad Prism 8.0.1.

No fixed fluorescence threshold was applied to define positivity. Instead, reactions were classified as positive based on a progressive, time-dependent increase in fluorescence intensity relative to concurrent negative controls (NC H_2_O and NC RPA), which remained at baseline throughout the 30-min reaction. All experiments were performed in triplicate; no false-positive or false-negative results were observed among the experimental replicates.

### 2.7. Sensitivity Analysis

The analytical sensitivity of the assay was evaluated using tenfold serial dilutions of genomic DNA. The lowest detectable concentration under the experimental conditions tested was defined as the lowest DNA concentration at which consistent amplification and a detectable increase in fluorescence signal were observed compared to the negative control.

Genomic DNA concentration was determined using a NanoDrop One spectrophotometer (Thermo Scientific, Waltham, MA, USA). Genome copy numbers were calculated from the measured DNA concentration assuming an average *A. baumannii* genome size of approximately 4.0 Mb using the standard molecular weight conversion formula. Tenfold serial dilutions were then prepared based on the calculated genome copy numbers.

$$\mathrm{Genome}\;\mathrm{copies}\;=\;\frac{\mathrm{DNA}\;\mathrm{mass}\left(ng\right)\;\times \;6.022\;\times \;10^{23}}{\mathrm{Genome}\;\mathrm{size}(bp)\;\times \;660\;\times \;10^{9}}$$

### 2.8. Specificity Analysis

The specificity of the assay was evaluated using genomic DNA from multiple bacterial species (*A. baumannii*, *A. pittii*, *A. nosocomialis*, *S. aureus*, *K. pneumoniae*, *P. aeruginosa*, *S. enterica*, *E. coli*, *S. agalactiae*, *E. ludwigii*, *S. haemolyticus*, and *A. junii*). The analysis was performed using the multiplex RPA-CRISPR/Cas12a system, followed by evaluation of fluorescence signal generation.

### 2.9. Evaluation of Clinical Isolates Using Multiplex RPA

To evaluate the analytical performance of the workflow, genomic DNA extracted from 63 clinical *A. baumannii* isolates was analyzed using the multiplex RPA–CRISPR/Cas12a system as described in Section 2.4 and Section 2.5. Fluorescence signals were monitored in real time using the CFX96 system (Bio-Rad) as described in Section 2.6.

Isolates were obtained from the clinical microbiology archive of the collaborating tertiary care hospital as part of routine diagnostic workflow. Species identification was confirmed by PCR targeting the intrinsic *bla_OXA-51_* gene, and amplification products were subjected to Sanger sequencing followed by BLAST (online NCBI BLASTn web service) analysis against the NCBI GenBank database, confirming the identity of all isolates as *A. baumannii*.

### 2.10. Statistical Analysis

All RFU data were visualized using GraphPad Prism 8.0.1 software and R statistical software [58,59]. To facilitate spatial comparison of the 64 samples, data were reorganized into an 8 × 8 coordinate matrix. The heatmap was generated using the geom_tile function within the ggplot2 library [60]. Fluorescence intensity was represented via a continuous scale ranging from light green (low fluorescence intensity) to dark green (high fluorescence intensity).

All experiments were performed in at least three independent replicates. Data are presented as mean ± standard deviation. All fluorescence measurements were performed in triplicate, and the coefficient of variation (CV) was calculated for each concentration and the negative controls.

## 3. Results

### 3.1. Multiplex RPA and CRISPR/Cas12a Detection of bla_OXA-23_ and bla_OXA-40_

**Figure 3:**
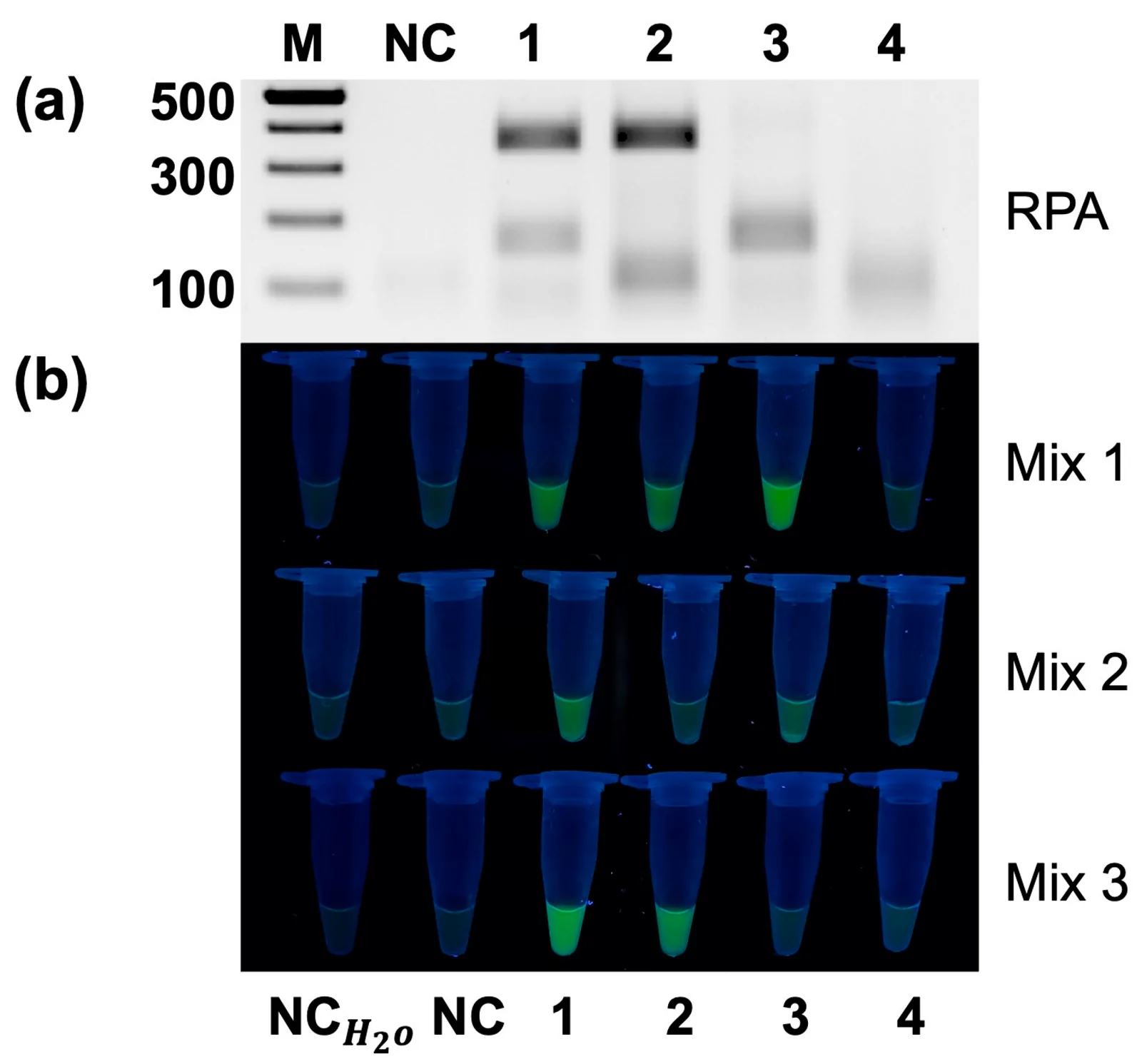
Multiplex RPA and CRISPR/Cas12a detection of *bla_OXA-23_* and *bla_OXA-40_*. (**a**) Agarose gel electrophoresis of RPA products. Lane M: DNA marker; NC: negative control (H_2_O); 1: genomic DNA containing both *bla_OXA-23_* and *bla_OXA-40_*; 2: genomic DNA containing *bla_OXA-23_* only; 3: genomic DNA containing *bla_OXA-40_* only; 4: genomic DNA lacking both target genes. (**b**) Fluorescence readout of CRISPR/Cas12a detection. Panels correspond to reactions with H_2_O, negative RPA product, and positive samples detected using Mix 1 (both crRNAs), Mix 2 (*bla_OXA-40_* crRNA), and Mix 3 (*bla_OXA-23_* crRNA).

To evaluate the feasibility of simultaneous detection of the *bla_OXA-23_* and *bla_OXA-40_* genes, multiplex RPA was performed followed by CRISPR/Cas12a detection (Figure 3). A single primer mix targeting both genes was used, while different genomic DNA samples served as templates.

Agarose gel electrophoresis demonstrated successful simultaneous amplification of both targets, producing distinct bands corresponding to the expected sizes (*bla_OXA-23_*—355 bp; *bla_OXA-40_*—157 bp). No amplification was observed in the negative control. Samples containing genomic DNA with both target genes produced two bands, whereas samples containing only *bla_OXA-23_* or *bla_OXA-40_* yielded the corresponding single bands. No amplification was detected in samples lacking both target genes, confirming the specificity of the assay.

Fluorescence analysis corroborated these findings. No signal was observed under control conditions, whereas strong fluorescence was detected in the presence of target amplicons. The use of different crRNA combinations showed that the mixture containing both crRNAs (Mix 1) generated a fluorescence signal in the presence of either target, whereas the individual crRNAs provided target-specific detection in separate reactions.

### 3.2. Sensitivity Analysis of bla_OXA-23_ Detection

**Figure 4:**
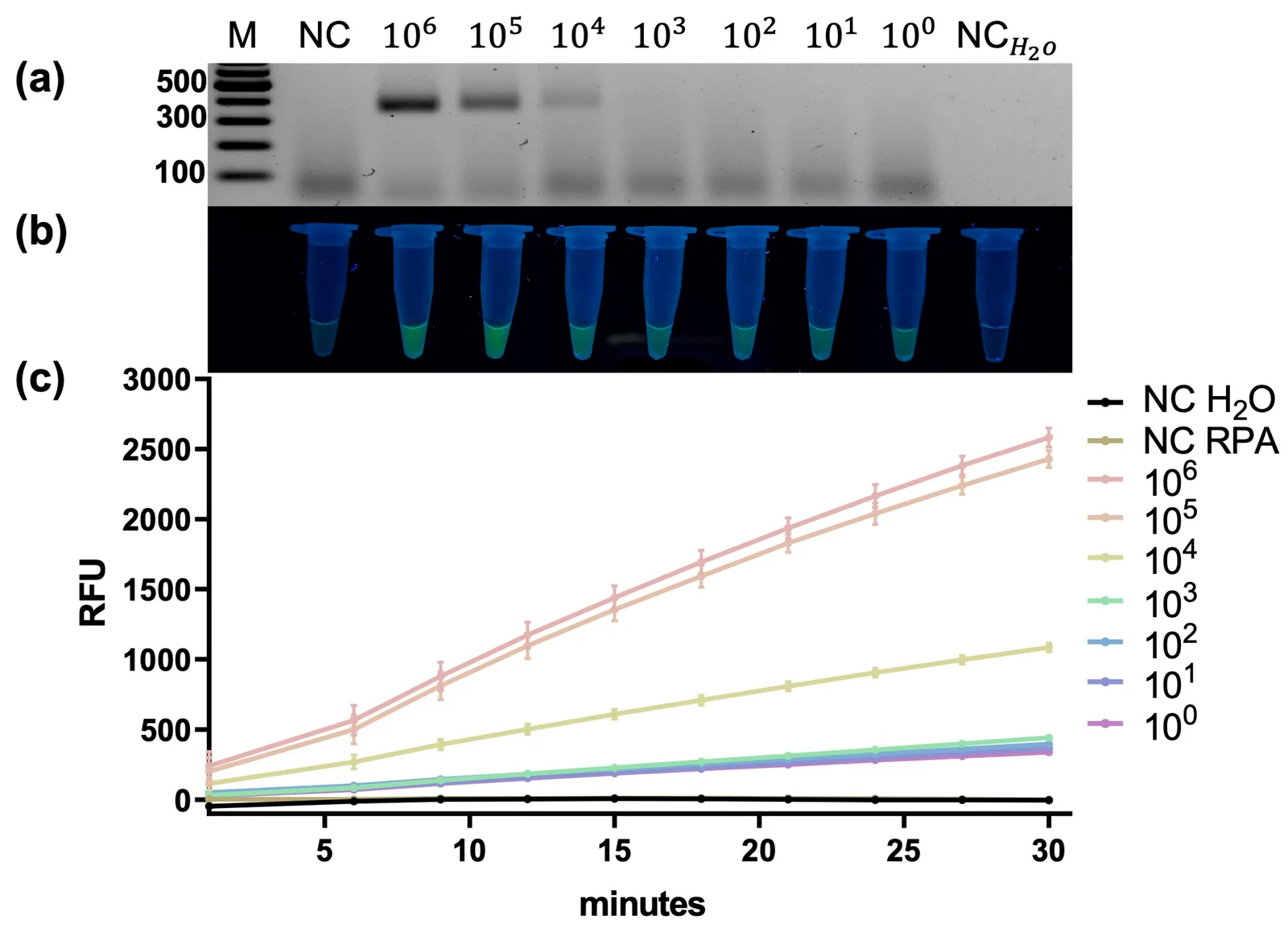
Sensitivity of *bla_OXA-23_* detection using multiplex RPA-CRISPR/Cas12a system. (**a**) Agarose gel electrophoresis of RPA products from tenfold serial dilutions of genomic DNA containing the *bla_OXA-23_* gene. Band intensity decreases with template concentration; amplification is detectable down to approximately 10^3^ copies. (**b**) Fluorescence readout of CRISPR/Cas12a detection for the same dilution series. Positive reactions remain distinguishable from the negative control even at near single-copy levels. (**c**) Real-time fluorescence curves corresponding to tenfold serial dilutions of genomic DNA. Individual curves are labeled according to the corresponding genome copy number shown in the figure. Positive samples exhibit a clear increase in fluorescence over time, whereas both negative controls remain at baseline.

The analytical sensitivity of blaOXA-23 detection was evaluated using tenfold serial dilutions of genomic DNA (Figure 4). RPA products were visually detectable by agarose gel electrophoresis down to a nominally calculated concentration of approximately 10^4^–10^3^ *A. baumannii* genome equivalents per reaction. In contrast, the CRISPR/Cas12a-based fluorescence readout remained distinguishable from the negative controls at the lowest tested DNA dilution, at which the RPA product was no longer visible by agarose gel electrophoresis. These results indicate that CRISPR/Cas12a fluorescence detection provided a more sensitive readout than gel-based visualization under the experimental conditions tested. Real-time fluorescence analysis further supported these findings by showing a distinct time-dependent increase in fluorescence in positive reactions compared with the negative controls, including at low template concentrations.

Triplicate fluorescence measurements showed low intra-assay coefficients of variation across the tested concentrations, supporting good intra-assay repeatability of the assay (Supplementary Table S1).

### 3.3. Sensitivity Analysis of bla_OXA-40_ Detection

**Figure 5:**
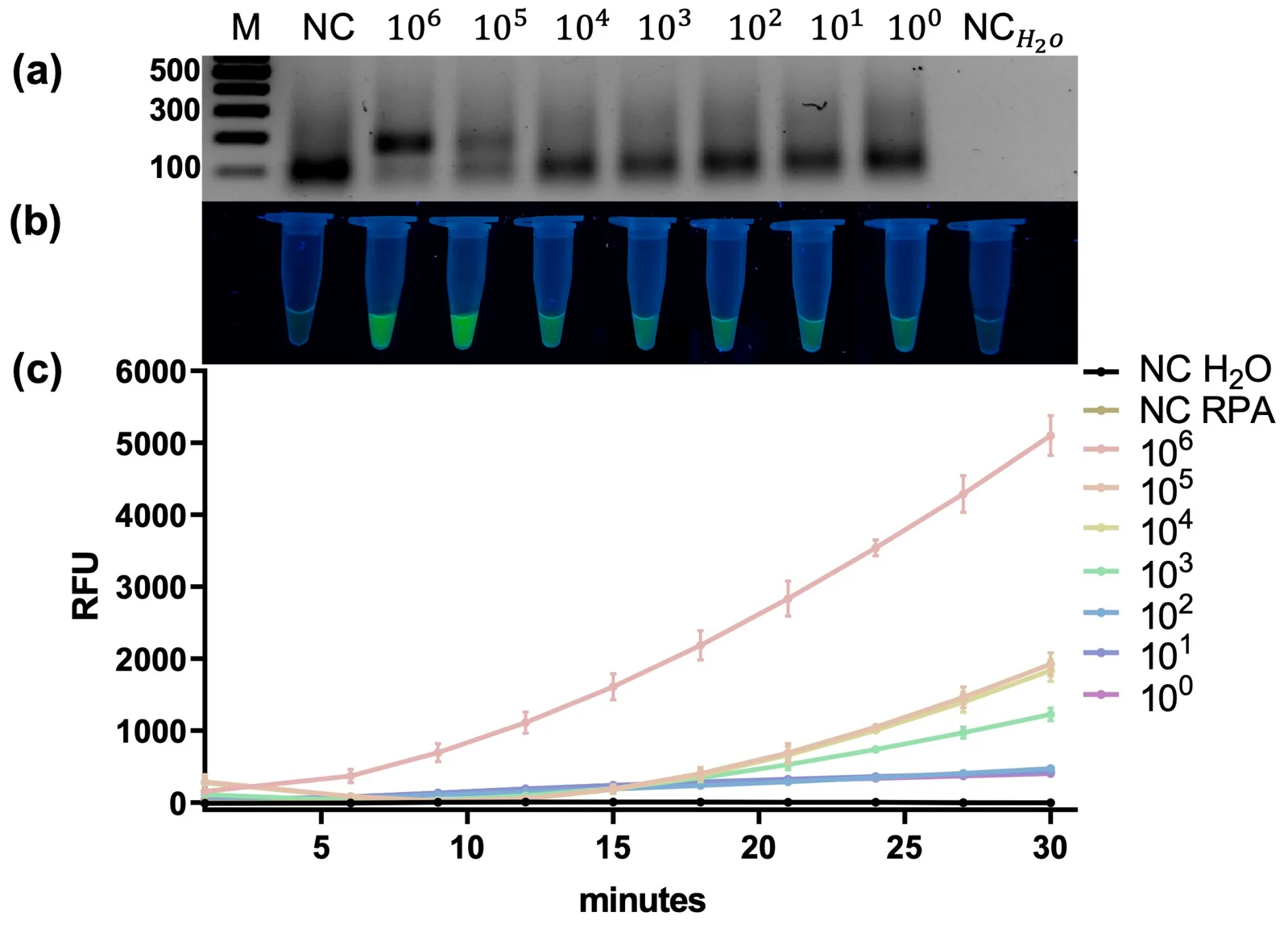
Sensitivity of *bla_OXA-40_* detection using multiplex RPA-CRISPR/Cas12a system. (**a**) Agarose gel electrophoresis of RPA products from tenfold serial dilutions of genomic DNA containing the *bla_OXA-40_* gene. Amplification is detectable down to approximately 10^5^ copies, indicating lower RPA sensitivity for this target compared to *bla_OXA-23_*. (**b**) Fluorescence readout of CRISPR/Cas12a detection for the same dilution series. Positive samples remain distinguishable from the negative control even at near single-copy levels. (**c**) Real-time fluorescence curves corresponding to tenfold serial dilutions of genomic DNA. Individual curves are labeled according to the corresponding genome copy number shown in the figure (10^6^–10^0^), together with the negative controls (NC H_2_O and NC RPA).

A similar analysis was performed for the *bla_OXA-40_* gene (Figure 5). Under RPA conditions, the visual detection limit was approximately 10^5^ copies, indicating lower amplification efficiency compared to *bla_OXA-23_*. However, CRISPR/Cas12a detection significantly improved sensitivity, allowing clear differentiation between positive and negative samples down to single-copy levels. These findings indicate that CRISPR/Cas12a effectively compensates for target-dependent limitations of RPA.

Triplicate fluorescence measurements showed low intra-assay coefficients of variation across the tested concentrations, supporting good intra-assay repeatability of the assay (Supplementary Table S2).

### 3.4. Sensitivity of Multiplex RPA Coupled to Pooled Dual-crRNA CRISPR/Cas12a Detection

**Figure 6:**
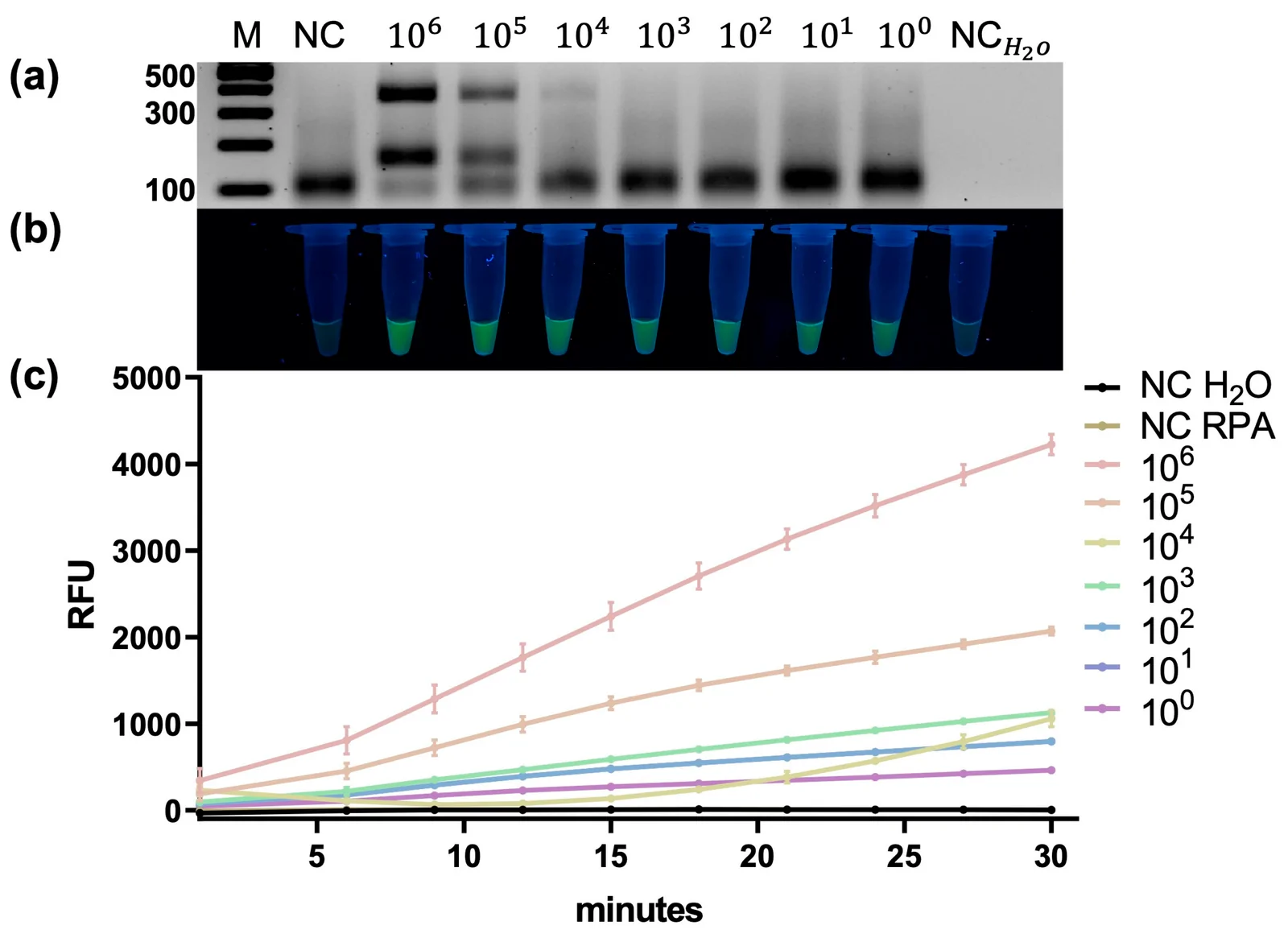
Sensitivity of multiplex *bla_OXA-23_* and *bla_OXA-40_* detection using the RPA-CRISPR/Cas12a system. (**a**) Agarose gel electrophoresis of RPA products from tenfold serial dilutions of genomic DNA containing both target genes. Visual detection limits are approximately 10^3^ copies for *bla_OXA-23_* and 10^5^ copies for *bla_OXA-40_*, reflecting differences in amplification efficiency under multiplex conditions. (**b**) Fluorescence readout of CRISPR/Cas12a detection for the same dilution series. Positive samples remain distinguishable from the negative control across all tested concentrations, including near single-copy levels. (**c**) Real-time fluorescence curves showing signal kinetics in multiplex format. Consistent separation between positive samples and the negative control confirms that multiplexing does not significantly compromise detection sensitivity.

Sensitivity analysis of the multiplex system (Figure 6) showed that the visual detection limits by RPA remained at approximately 10^3^ copies for *bla_OXA-23_* and 10^5^ copies for *bla_OXA-40_*. Despite differences in amplification efficiency, CRISPR/Cas12a fluorescence detection enabled reliable discrimination between positive and negative samples at single-copy template concentrations for both targets under the experimental conditions tested. These results demonstrate that multiplexing does not significantly compromise the sensitivity of CRISPR-based detection.

Triplicate fluorescence measurements showed low intra-assay coefficients of variation across the tested concentrations, supporting good intra-assay repeatability of the assay (Supplementary Table S3).

### 3.5. Specificity Analysis

**Figure 7:**
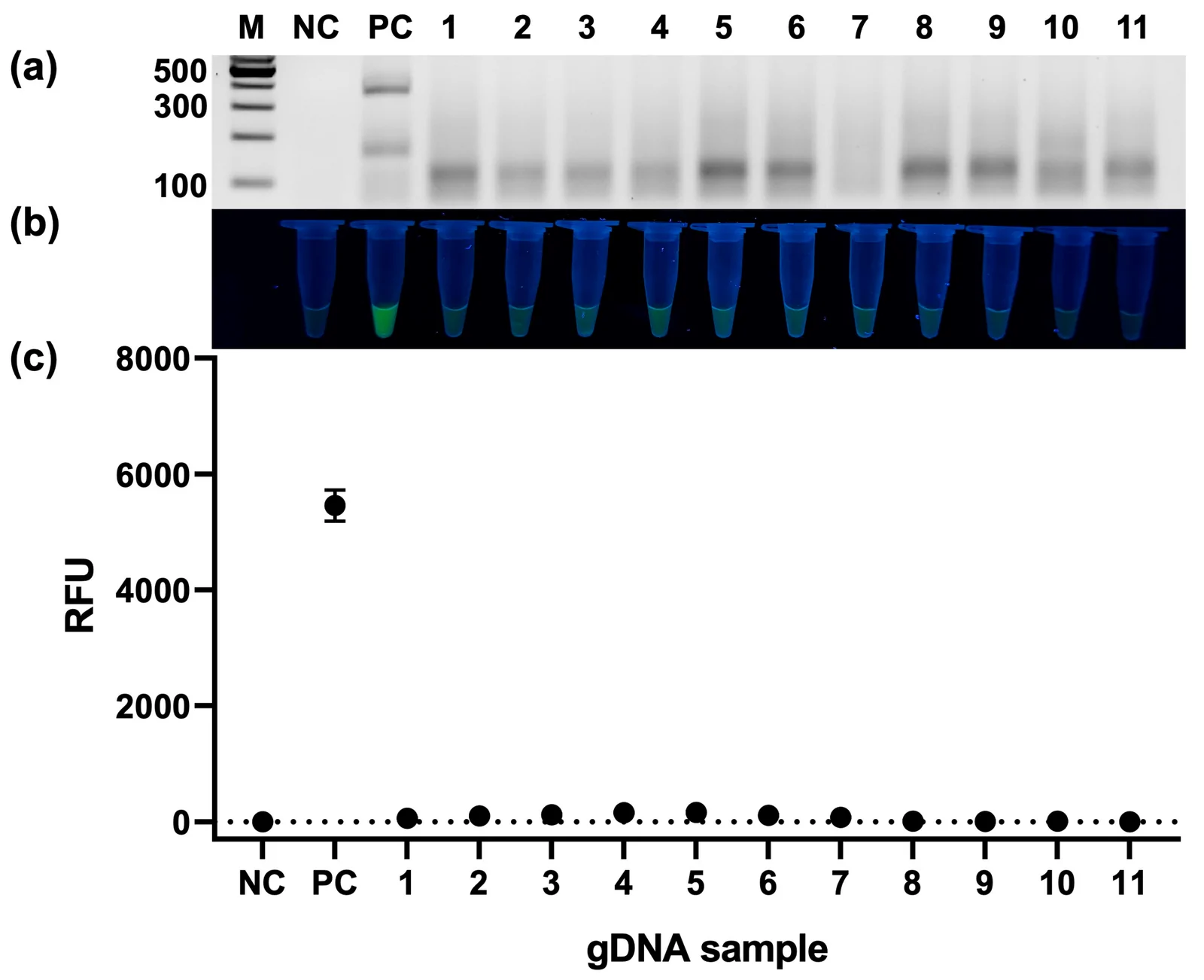
Specificity analysis of the multiplex RPA-CRISPR/Cas12a system for detection of *bla_OXA-23_* and *bla_OXA-40_*. (**a**) Agarose gel electrophoresis of RPA products from genomic DNA of different bacterial species. Specific amplification is observed only in the positive control (*A. baumannii*), with no amplification in non-target species or the negative control (H_2_O). (**b**) Fluorescence readout of CRISPR/Cas12a detection for the same sample panel. A strong signal is detected exclusively in the positive control; all other samples remain at baseline. (**c**) Bar chart of quantitative fluorescence intensity. Elevated signal is observed only for *A. baumannii*, while all tested species—1: *S. aureus*; 2: *E. coli*; 3: *P. aeruginosa*; 4: *S. enterica*; 5: *K. pneumoniae*; 6: *S. agalactiae*; 7: *A. nosocomialis*; 8: *E. ludwigii*; 9: *S. haemolyticus*; 10: *A. pittii*; 11: *A. junii*—remain at baseline. NC: negative control (H_2_O); PC: positive control (*A. baumannii*).

The specificity of the developed assay was evaluated using genomic DNA from various bacterial species (Figure 7). A fluorescence signal was observed exclusively in the positive control (*A. baumannii* containing both target genes), whereas all other tested species (*S. aureus*, *E. coli*, *P. aeruginosa*, *S. enterica*, *K. pneumoniae*, *S. agalactiae*, *A. nosocomialis*, *E. ludwigii*, *S. haemolyticus*, *A. pittii*, and *A. junii*) showed no signal or fluorescence comparable to the negative control. These results confirm the high specificity of both RPA and CRISPR/Cas12a detection, as well as the absence of cross-reactivity.

### 3.6. Detection of Clinical Isolates

**Figure 8:**
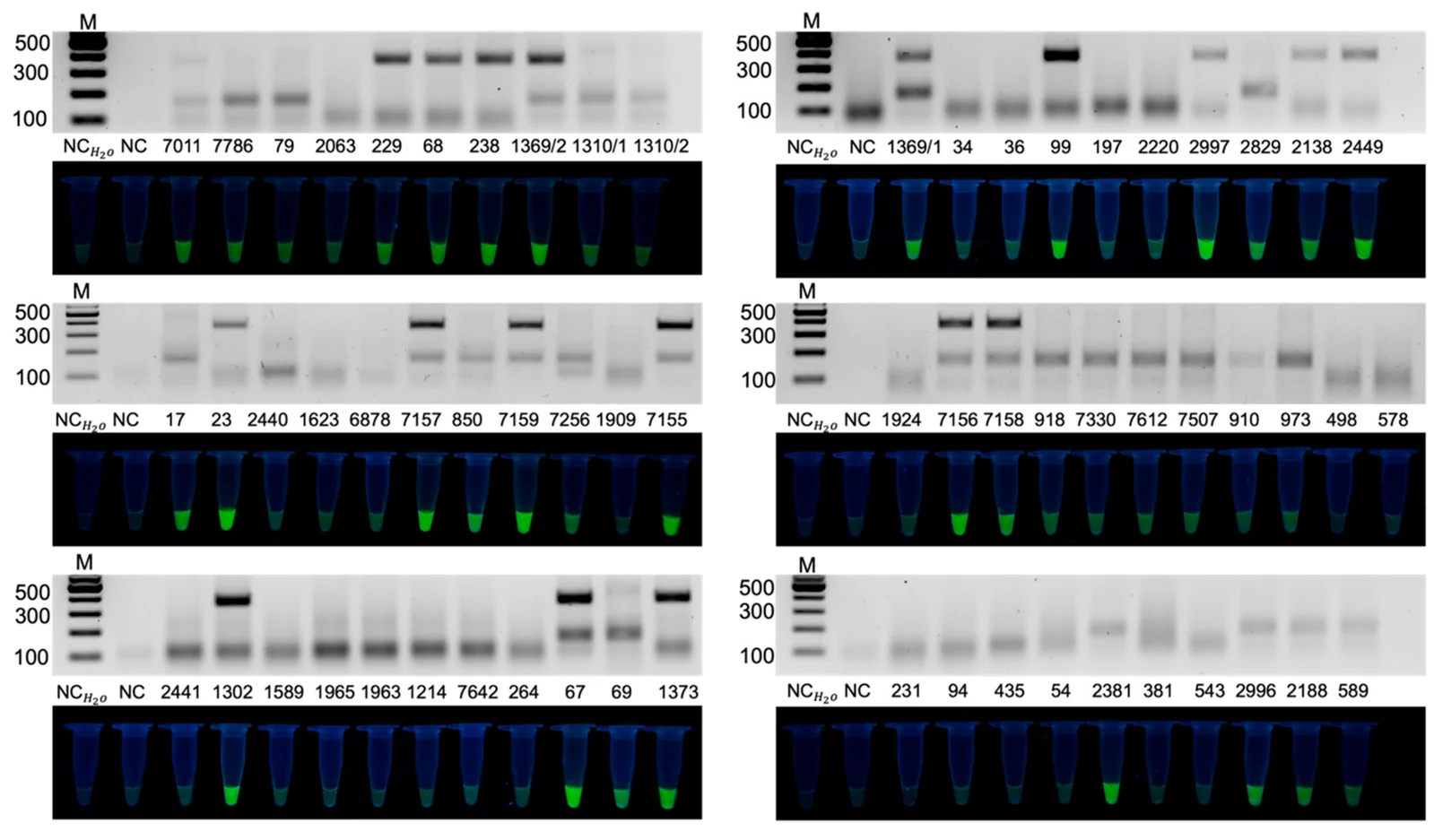
Detection of *bla_OXA-23_* and *bla_OXA-40_* in purified genomic DNA extracted from clinical *A. baumannii* isolates using the multiplex RPA-CRISPR/Cas12a system. Agarose gel electrophoresis of multiplex RPA products (**upper panels**) and corresponding endpoint fluorescence images of CRISPR/Cas12a reactions (**lower panels**) are shown for representative clinical isolates. Green fluorescence under blue-light illumination indicates positive detection of the target genes. The **left** lane of each gel contains the DNA molecular weight marker (M).

The developed method was evaluated using purified genomic DNA extracted from 63 clinical *A. baumannii* isolates (Figure 8). The results showed that the *bla_OXA-23_* gene was detected in 19 isolates (30.2%), the *bla_OXA-40_* gene was detected in 28 isolates (44.4%), while the simultaneous presence of both genes was detected in 9 samples (14.3%). At least one target gene was identified in 38 of 63 isolates (60.3%). The data obtained demonstrate the feasibility of the method for analysis of purified genomic DNA from cultured clinical isolates and reflect the prevalence of blaOXA class carbapenemases among the studied isolates. The RPA results fully correlated with the CRISPR/Cas12a detection data and were confirmed by fluorescence analysis. These results demonstrated concordance between the RPA and CRISPR/Cas12a readouts.

**Figure 9:**
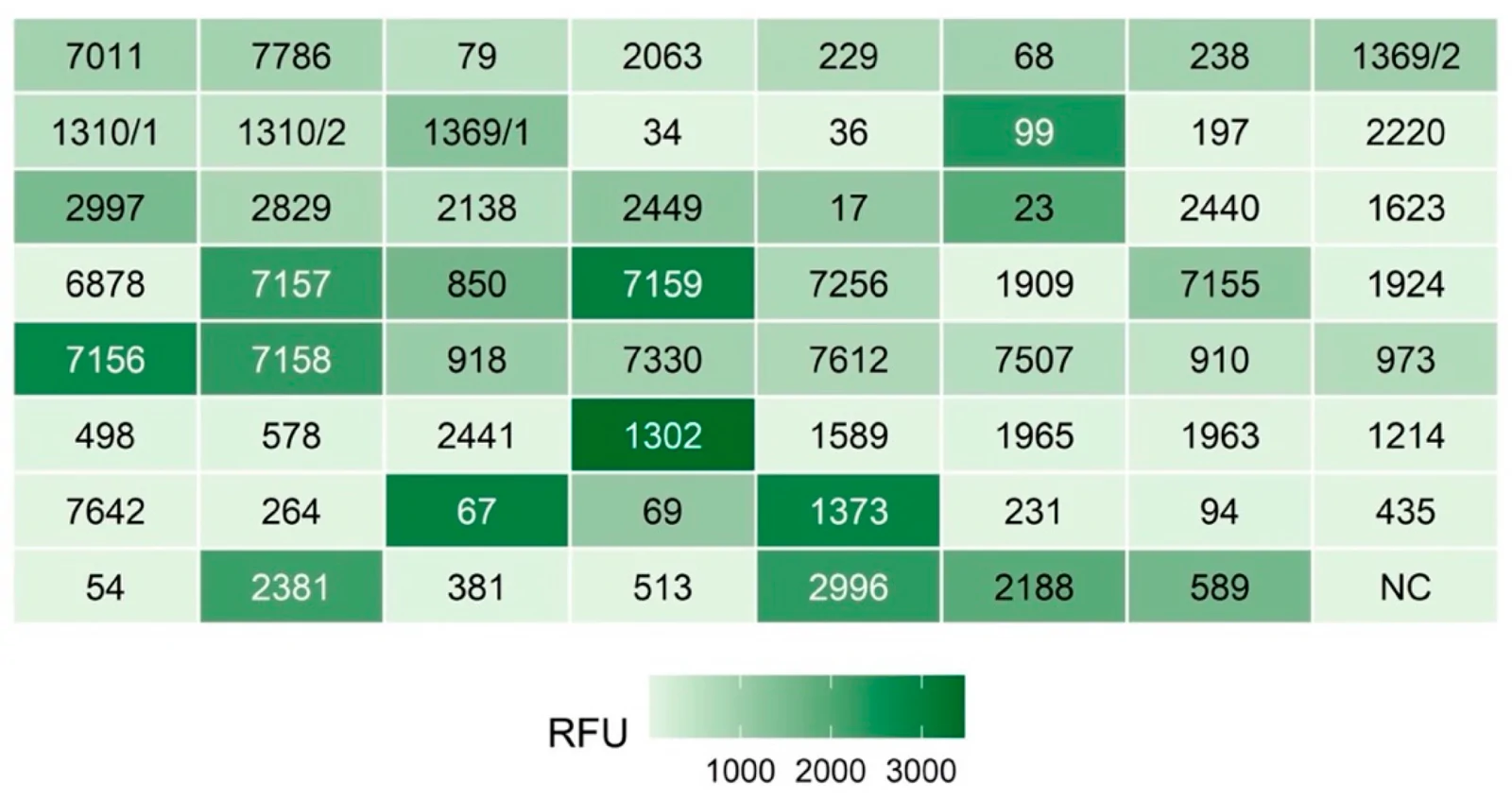
Heatmap representation of endpoint fluorescence intensity (RFU) across all tested clinical isolates. The color scale ranges from 1000 to 3000 RFU, where light green indicates lower fluorescence intensity and dark green indicates higher fluorescence intensity. Numbers within the cells correspond to the measured endpoint RFU values. NC, negative control.

**Figure 10:**
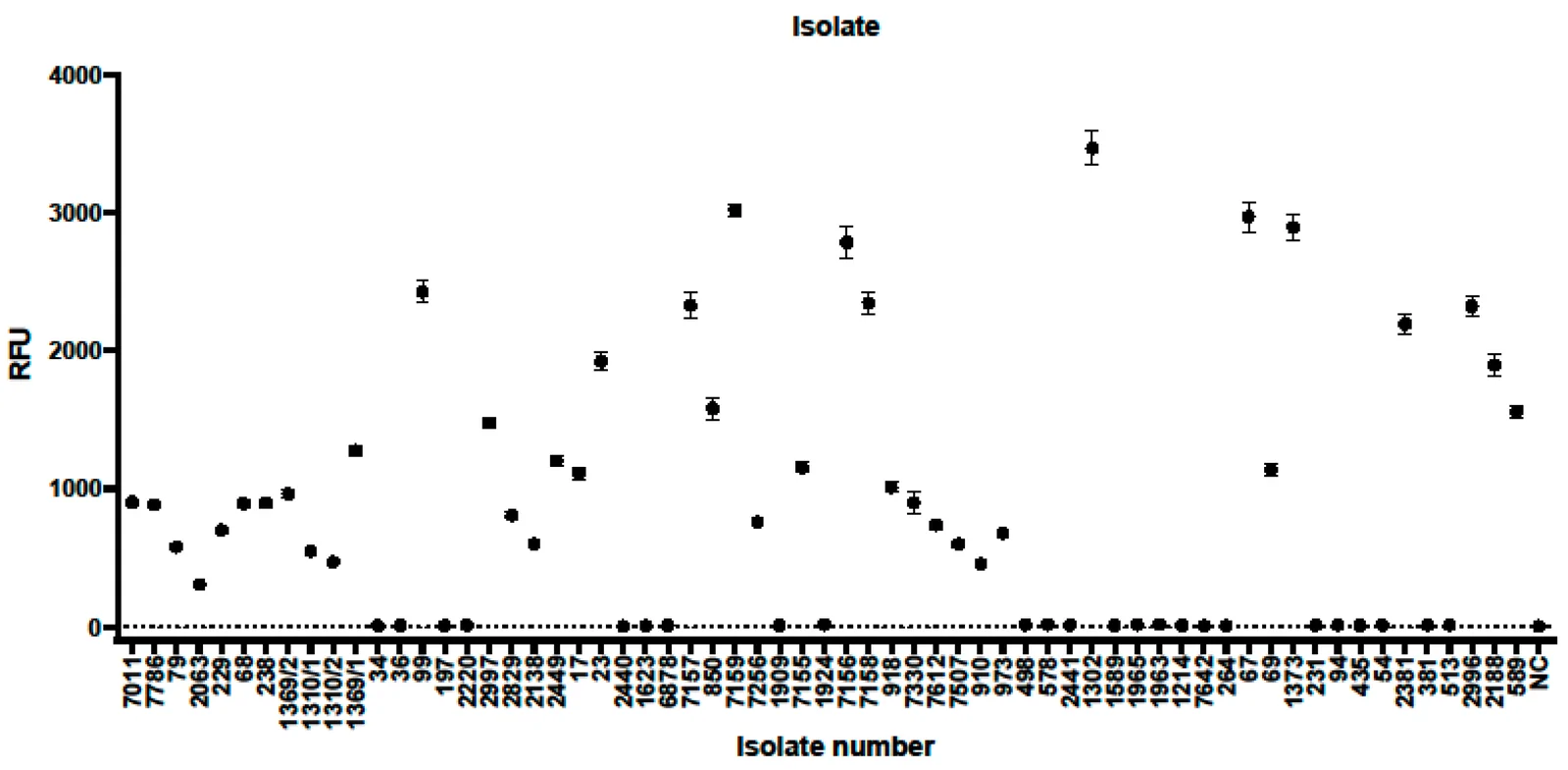
Quantitative analysis of endpoint fluorescence signals obtained from clinical isolates using the multiplex RPA-CRISPR/Cas12a system. Endpoint fluorescence values are presented as mean ± standard deviation (SD) in relative fluorescence units (RFU). Each data point corresponds to an individual clinical isolate. The dashed horizontal line indicates the baseline fluorescence level of the negative control (NC).

To further visualize the distribution and quantitative characteristics of fluorescence signals obtained from clinical isolates, a heatmap and corresponding statistical analysis were generated (Figure 9 and Figure 10). The heatmap (Figure 9) illustrates the fluorescence intensity patterns across all tested isolates, clearly distinguishing samples positive for *bla_OXA-23_*, *bla_OXA-40_*, or both targets, as well as negative samples with baseline signal levels.

## 4. Discussion

The present study describes the development and preliminary analytical evaluation of a multiplex two-step RPA–CRISPR/Cas12a assay designed to assess the feasibility of simultaneously using two target-specific crRNAs in a single CRISPR/Cas12a reaction. The unified assay generated a detectable fluorescence signal in the presence of *bla*_OXA-23_, *bla*_OXA-40_, or both resistance genes in purified genomic DNA extracted from cultured clinical *A. baumannii* isolates. Under the experimental conditions tested, positive signals were observed at nominal template concentrations corresponding to approximately one genome copy per reaction, and no cross-reactivity was observed with the tested panel of non-target bacterial species. Evaluation using genomic DNA from 63 cultured clinical isolates further supported the analytical feasibility of the approach. However, the study was conceived as a proof-of-concept analytical feasibility assessment rather than the development or formal clinical validation of a complete diagnostic workflow. Further studies using primary clinical specimens, standardized sample preparation procedures, and formal diagnostic performance criteria will be required before potential point-of-care application can be considered.

A major finding of this work is the successful implementation of multiplex RPA without significant loss of amplification efficiency. Both target genes were efficiently co-amplified in a single reaction using two primer pairs under isothermal conditions at 37 °C for 15 min, yielding distinct and specific products. Despite the inherent challenges of multiplex isothermal amplification—including primer competition, limited options for temperature-based optimization, and the absence of thermal cycling stringency—the robustness of the primer design ensured reliable co-amplification across a range of template concentrations. This result confirms that the selected reaction conditions are suitable for simultaneous amplification of two genetically distinct resistance determinants without mutual interference.

Sensitivity analysis revealed target-dependent differences in amplification efficiency at the RPA stage. The visual detection limit for *bla_OXA-23_* was approximately 10^3^ copies, whereas for *bla_OXA-40_* it was approximately 10^5^ copies under RPA alone, indicating lower intrinsic amplification efficiency for the latter target. These differences are likely attributable to variations in sequence composition, GC content, secondary structure formation, or primer–template interaction kinetics. Critically, the subsequent CRISPR/Cas12a detection step, performed in a single reaction vessel using two target-specific crRNAs at 37 °C for 30 min, effectively compensated for these differences, enabling reliable fluorescence-based detection down to single-copy levels for both *bla_OXA-23_* and *bla_OXA-40_*. This finding underscores the essential role of Cas12a-mediated collateral cleavage as a signal amplification mechanism that overcomes the inherent sensitivity limitations of RPA, particularly in multiplex formats where amplification efficiency may vary between targets. Importantly, sensitivity analysis of the fully multiplexed system confirmed that multiplexing does not significantly compromise CRISPR-based detection performance, demonstrating that single-copy sensitivity is retained when both targets are processed simultaneously. This outcome is particularly noteworthy given that the simultaneous use of two crRNAs within a single CRISPR/Cas12a reaction is conventionally regarded as technically challenging owing to potential competition between crRNAs and the associated risk of reduced analytical sensitivity; in the present system; however, this challenge is effectively circumvented, reframing dual-crRNA CRISPR/Cas12a detection as an advantage rather than a constraint. The clinical rationale for this dual-target design is further reinforced by the fact that infections caused by *A. baumannii* strains harboring either *bla_OXA-23_* or *bla_OXA-40_* share identical clinical management and require analogous therapeutic strategies aimed at overcoming carbapenem resistance, rendering their simultaneous identification in a single assay both analytically justified and clinically warranted.

It should be noted that the sensitivity figures reported here reflect the lowest template concentration reliably distinguishable from background under the specific experimental conditions tested, rather than a formal limit of detection (LoD) established according to CLSI EP17 or equivalent analytical validation guidelines (e.g., LoD defined at a 95% detection probability across independent replicates). Formal LoD determination, together with intra- and inter-assay precision studies across a wider range of operators and reagent lots, will be addressed in future validation work.

Real-time fluorescence analysis provided additional insight into signal generation kinetics. Amplification curves clearly distinguished positive samples from negative controls across a broad range of template concentrations, enabling not only qualitative but also semi-quantitative assessment of target presence. This capability provides an internal quality control mechanism that reduces the likelihood of false-negative results due to reaction failure, and expands the diagnostic information content of the system beyond simple endpoint detection.

The specificity of the developed system was evaluated against a panel of eleven clinically relevant bacterial species, including common nosocomial pathogens—*Staphylococcus aureus*, *Escherichia coli*, *Pseudomonas aeruginosa*, *Salmonella enterica*, *Klebsiella pneumoniae*, *Streptococcus agalactiae*, *Enterobacter ludwigii*, and *Staphylococcus haemolyticus*—as well as three phylogenetically closely related non-baumannii *Acinetobacter* species: *A. nosocomialis*, *A. pittii*, and *A. junii*. The inclusion of non-baumannii *Acinetobacter* species is of particular clinical importance, as misidentification within the *Acinetobacter calcoaceticus–baumannii* complex remains a recognized challenge in routine microbiology. The complete absence of cross-reactivity across all eleven tested species, including the closely related *Acinetobacter* spp., confirms the high specificity of the crRNA design and validates the suitability of the system for application in clinical settings where mixed microbial flora may be present.

The developed system was evaluated using genomic DNA extracted from 63 cultured clinical *Acinetobacter baumannii* isolates obtained from a tertiary care hospital. Species identification was confirmed by PCR targeting the intrinsic *bla_OXA-51_* gene, and amplification products were subjected to Sanger sequencing followed by BLAST analysis against the NCBI GenBank database, confirming the identity of all isolates as *A. baumannii*. Among the 63 isolates, 38 (60.3%) carried at least one of the two target resistance genes. Specifically, *bla_OXA-23_* was detected in 19 isolates (30.2%), *bla_OXA-40_* in 28 isolates (44.4%), and co-carriage of both genes was identified in 9 isolates (14.3%). The remaining 25 isolates (39.7%) were negative for both target genes, likely harboring alternative carbapenem resistance mechanisms such as OXA-58-like enzymes, porin loss, or efflux pump overexpression. Collectively, these results confirm that the developed system is capable of detecting the predominant acquired class D carbapenemases in the majority of CRAB clinical isolates within a single 45-min workflow, providing clinically actionable information without the need for specialized laboratory infrastructure.

The resistance mechanisms present in the isolates negative for both *bla_OXA-23_* and *bla_OXA-40_* were not investigated in the present study. These isolates may carry alternative carbapenem resistance determinants, such as OXA-58-like enzymes, metallo-β-lactamases, or non-enzymatic resistance mechanisms, which warrant further investigation.

The present study evaluated the assay using purified genomic DNA extracted from cultured clinical isolates rather than directly from primary clinical specimens. Therefore, the reported workflow includes bacterial isolation and genomic DNA extraction prior to RPA-CRISPR/Cas12a analysis. Future studies should evaluate the performance of the assay using DNA extracted directly from clinical specimens and optimize sample preparation for true point-of-care applications.

The dual-target design of the developed system addresses a critical gap in current diagnostic approaches for CRAB. By simultaneously targeting *bla_OXA-23_* and *bla_OXA-40_*—the two most prevalent acquired class D carbapenemases in *A. baumannii* globally and in Kazakhstan specifically—the system provides broader coverage of clinically relevant carbapenem resistance than any single-target assay. The high proportion of isolates carrying at least one of the two target genes (60.3%) confirms the epidemiological relevance of this target selection in the local clinical context.

The present assay can be compared with two recently published RPA-CRISPR/Cas12a platforms for carbapenem-resistant organisms. Zhou et al. [55] developed a multiplex RPA-CRISPR/Cas12a system targeting the intrinsic species marker *bla_OXA-51_* together with the acquired carbapenemase gene *bla_OXA-23_*, achieving a mass-based detection limit of 1.3 × 10^−6^ ng/µL and 100% concordance with PCR across 30 clinical isolates within 90 min. In contrast, the present assay targets two distinct acquired class D carbapenemase genes (*bla_OXA-23_* and *bla_OXA-40_*) rather than combining a species marker with a single resistance determinant, using a single-tube CRISPR/Cas12a step with two gene-specific crRNAs in the same reaction and achieving nominal calculated concentration corresponding to approximately one genome copy per reaction for each target, on a larger clinical panel (63 isolates) within a shorter 45-min RPA-CRISPR workflow. Yang et al. [56] developed an RPA-coupled CRISPR/Cas12a platform (RCCS) for *bla*KPC and *bla*NDM in carbapenem-resistant Enterobacterales, completing the assay within 50 min including 10 min of DNA extraction (20 min RPA plus 10–20 min CRISPR/Cas12a). Direct comparison of overall turnaround time between this platform and the present assay is not straightforward, since the reported 45-min duration of the present assay reflects the RPA-CRISPR/Cas12a reaction time only, performed on previously extracted genomic DNA, and does not include the bacterial culture and DNA extraction steps discussed above. A harmonized head-to-head comparison of sensitivity, specificity, and total turnaround time (including sample preparation) across these platforms was not performed in this study, as each assay was validated using different sample panels, target genes, and reference methods; such a comparison is a priority for future work.

The analytical workflow evaluated in the present study consisted of 15 min of multiplex RPA followed by 30 min of CRISPR/Cas12a detection, resulting in a total assay time of approximately 45 min following genomic DNA extraction. The reported workflow does not include bacterial culture or DNA extraction, as the assay was evaluated using purified genomic DNA extracted from cultured clinical isolates. Consequently, additional studies are required to optimize sample preparation and evaluate assay performance directly on clinical specimens prior to point-of-care implementation.

Overall, the developed multiplex RPA-CRISPR/Cas12a system represents a robust, rapid, and clinically relevant diagnostic platform for the simultaneous detection of the predominant acquired carbapenemase genes in *A. baumannii*. Its high analytical performance, combined with operational simplicity and point-of-care compatibility, positions it as a valuable tool for timely identification of CRAB in both clinical and surveillance settings.

## 5. Conclusions

In this study, a multiplex RPA followed by pooled dual-crRNA CRISPR/Cas12a screening was developed for indicating the presence of blaOXA-23 and/or blaOXA-40 in purified genomic DNA extracted from cultured clinical *A. baumannii* isolates. The system demonstrated detection of single-copy template concentrations under the experimental conditions evaluated for both targets, complete specificity against eleven clinically relevant bacterial species including closely related non-baumannii *Acinetobacter* spp., and demonstrated reliable performance across 63 clinical isolates, with results concordant with PCR-based reference genotyping. The high proportion of clinical isolates carrying at least one of the two target genes (60.3%), with *bla_OXA-40_* predominating (44.4%) followed by *bla_OXA-23_* (30.2%), confirms that *bla_OXA-23_* and *bla_OXA-40_* represent the dominant acquired carbapenem resistance determinants in *A. baumannii* circulating in the tertiary care setting, underscoring the epidemiological relevance and local clinical utility of the selected target combination. The entire diagnostic workflow was completed within 45 min at 37 °C without specialized laboratory equipment, combining 15 min of multiplexed isothermal RPA with 30 min of pooled dual-crRNA CRISPR/Cas12a fluorescence readout indicating the presence of at least one target in a single reaction vessel. By simultaneously targeting the two predominant acquired class D carbapenemases in *A. baumannii*, the system provides comprehensive coverage of clinically relevant carbapenem resistance within a single analytical pipeline. These characteristics position the developed platform as a promising tool for rapid, sensitive, and specific point-of-care detection of carbapenem-resistant *A. baumannii* in clinical and resource-limited settings.

## Data Availability

Data are contained within the article and Supplementary Materials.

## Supplementary Materials

The following supporting information can be downloaded at: https://www.mdpi.com/article/10.3390/bios16080422/s1, Table S1: Repeatability of fluorescence measurements for *bla_OXA-23_* detection; Table S2: Repeatability of fluorescence measurements for *bla_OXA-40_* detection; Table S3: Repeatability of fluorescence measurements for multiplex RPA–CRISPR/Cas12a detection of *bla_OXA-23_* and *bla_OXA-40_*.
