# Supplementary material for: Multiplex RPA-CRISPR/Cas12a Assay for Rapid Detection of Class D OXA-Type Carbapenem-Resistant Acinetobacter baumannii
Source: Biosensors (Basel). 2026 Aug 5;16(8):422. doi: 10.3390/bios16080422 (PMC13511001; doi:10.3390/bios16080422)
Supplement: Supplementary file 1 [file biosensors-16-00422-s001.zip › biosensors-4444716-supplementary.pdf]

biosensors MDPI

Supplementary Materials

Multiplex RPA-CRISPR/Cas12a Assay for Rapid Detection of Class D OXA-Type Carbapenem-Resistant *Acinetobacter baumannii*

Meruyert Amanzholova [1,2,†], Ainur Akimbekova [1,†], Aisha Shaizadinova [1,†], Nazgul Sutimbekova [3], Nelya Bissenova [4], Pavel Tarlykov [1] and Sailau Abeldenov [1,*]

[1] National Center for Biotechnology, Astana 010000, Kazakhstan
[2] Faculty of Biology and Biotechnology, Farabi Kazakh National University, Almaty 010000, Kazakhstan
[3] Department of Microbiology and Virology, Astana Medical University, Astana 010000, Kazakhstan
[4] National Scientific Medical Center, Astana 010000, Kazakhstan
* Correspondence: abeldenov@biocenter.kz
† These authors contributed equally to this work.

Table S1. Repeatability of fluorescence measurements for *bla*$_{OXA\text{-}23}$ detection.

	Mean	SD	CV (%)
NC H2O	-0.77	2.04	NA
NC RPA	4.59	2.11	NA
10^6	2381.17	69.03	2.90
10^5	2239.58	62.63	2.80
10^4	995.57	31.29	3.14
10^3	397.87	14.21	3.57
10^2	359.95	11.42	3.17
10^1	333.68	10.19	3.05
10^0	310.54	8.95	2.88

Values represent endpoint fluorescence measurements from three technical replicates.

Table S2. Repeatability of fluorescence measurements for *bla*$_{OXA\text{-}40}$ detection.

	Mean	SD	CV (%)
NC H2O	-0.46	2.47	NA
NC RPA	4.48	1.30	NA
10^6	4290.98	257.82	6.01
10^5	1461.20	146.18	10.00
10^4	1395.83	139.14	9.97
10^3	972.79	79.63	8.19
10^2	406.51	20.90	5.14
10^1	395.67	9.97	2.52
10^0	369.32	10.90	2.95

Values represent endpoint fluorescence measurements from three technical replicates.

Table S3. Repeatability of fluorescence measurements for multiplex RPA–CRISPR/Cas12a detection of *bla*$_{OXA\text{-}23}$ and *bla*$_{OXA\text{-}40}$.

	**Mean**	**SD**	**CV (%)**
NC H2O	7.33	2.12	NA
NC RPA	7.24	2.57	NA
10^6	3874.01	118.51	3.06
10^5	1919.10	49.96	2.60
10^4	794.53	81.23	10.22
10^3	1028.36	32.63	3.17
10^2	733.70	19.11	2.60
10^1	795.72	21.28	2.67
10^0	424.31	13.23	3.12

Values represent endpoint fluorescence measurements from three technical replicates.

Disclaimer/Publisher's Note: The statements, opinions and data contained in all publications are solely those of the individual author(s) and contributor(s) and not of MDPI and/or the editor(s). MDPI and/or the editor(s) disclaim responsibility for any injury to people or property resulting from any ideas, methods, instructions or products referred to in the content.

https://doi.org/10.3390/bios16080422
